# Latency Antigen α-Crystallin Based Vaccination Imparts a Robust Protection against TB by Modulating the Dynamics of Pulmonary Cytokines

**DOI:** 10.1371/journal.pone.0018773

**Published:** 2011-04-18

**Authors:** Bappaditya Dey, Ruchi Jain, Aparna Khera, Umesh D. Gupta, V. M. Katoch, V. D. Ramanathan, Anil K. Tyagi

**Affiliations:** 1 Department of Biochemistry, University of Delhi South Campus, New Delhi, India; 2 National JALMA Institute for Leprosy & Other Mycobacterial Diseases, Agra, Uttar Pradesh, India; 3 Department of Clinical Pathology, Tuberculosis Research Center, Chennai, Tamil Nadu, India; Statens Serum Institute, Denmark

## Abstract

**Background:**

Efficient control of tuberculosis (TB) requires development of strategies that can enhance efficacy of the existing vaccine *Mycobacterium bovis* Bacille Calmette Guerin (BCG). To date only a few studies have explored the potential of latency-associated antigens to augment the immunogenicity of BCG.

**Methods/Principal Findings:**

We evaluated the protective efficacy of a heterologous prime boost approach based on recombinant BCG and DNA vaccines targeting α-crystallin, a prominent latency antigen. We show that “rBCG prime - DNA boost” strategy (R/D) confers a markedly superior protection along with reduced pathology in comparison to BCG vaccination in guinea pigs (565 fold and 45 fold reduced CFU in lungs and spleen, respectively, in comparison to BCG vaccination). In addition, R/D regimen also confers enhanced protection in mice. Our results in guinea pig model show a distinct association of enhanced protection with an increased level of interleukin (IL)12 and a simultaneous increase in immuno-regulatory cytokines such as transforming growth factor (TGF)β and IL10 in lungs. The T cell effector functions, which could not be measured in guinea pigs due to technical limitations, were characterized in mice by multi-parameter flow cytometry. We show that R/D regimen elicits a heightened multi-functional CD4 Th1 cell response leading to enhanced protection.

**Conclusions/Significance:**

These results clearly indicate the superiority of α-crystallin based R/D regimen over BCG. Our observations from guinea pig studies indicate a crucial role of IL12, IL10 and TGFβ in vaccine-induced protection. Further, characterization of T cell responses in mice demonstrates that protection against TB is predictable by the frequency of CD4 T cells simultaneously producing interferon (IFN)γ, tumor necrosis factor (TNF)α and IL2. We anticipate that this study will not only contribute toward the development of a superior alternative to BCG, but will also stimulate designing of TB vaccines based on latency antigens.

## Introduction

The ability of *Mycobacterium tuberculosis* to survive the hostile immune responses and persist in a non-replicating dormant state is fundamental to its success as a human pathogen. One third of the world's population is asymptomatically infected with *M. tuberculosis* and represents an enormous pool of latent tuberculosis (TB) infections. Reactivation of these latent infections, which is responsible for majority of TB cases, contributes significantly to the problems associated with the incidence, transmission and pathogenesis of TB [1]. *M. bovis* Bacille Calmette Guerin (BCG), which provides consistent protection against severe forms of child hood TB, loses its potency gradually resulting in an inadequate protection in adults and elderly people [2]. Thus, for an efficient global control of TB, we require to develop new vaccination strategies to improve and sustain the protective efficacy of BCG.

Transcriptome and proteome analysis of *M. tuberculosis* revealed that α-crystallin (*acr, Rv2031c*), a member of DosR-DosS/DosT dormancy regulon, represents one of the most abundantly produced proteins during exposure to hypoxia, nutrient starvation and transition of actively dividing bacilli to a dormant state [3], [4]. Besides, latently infected individuals (healthy PPD+ and household contacts) exhibit increased lympho-proliferative and IFNγ response to α-crystallin as compared to patients with active TB [5]. These observations signify a crucial role of α-crystallin in the elicitation of protective immune responses and maintenance of a disease free state in these subjects, thus, making this antigen an attractive target for the development of new TB vaccines.

We have previously reported a DNA vaccine expressing α-crystallin (DNAacr) that protected guinea pigs against *M. tuberculosis* infection, however, it could not surpass the protective efficacy of BCG [6]. Thus, in the present study, we have developed an rBCG strain over-expressing α-crystallin (rBCGacr) and evaluated its protective efficacy in guinea pigs either alone or in combination with DNAacr in a heterologous prime boost regimen against *M. tuberculosis* challenge. In addition, we attempted to establish an association between the dynamic changes in the pulmonary cytokine milieu and vaccine-induced protection. Further, we delineated an association between multi-functional T cell responses and protective efficacy of these vaccine regimens. This study shows that vaccination regimens designed to target latency-associated antigens can elicit a superior protective immunity than BCG against TB.

## Results

### Analysis of expression and *in vitro* growth of rBCG strain over-expressing α-crystallin

**Figure 1:**
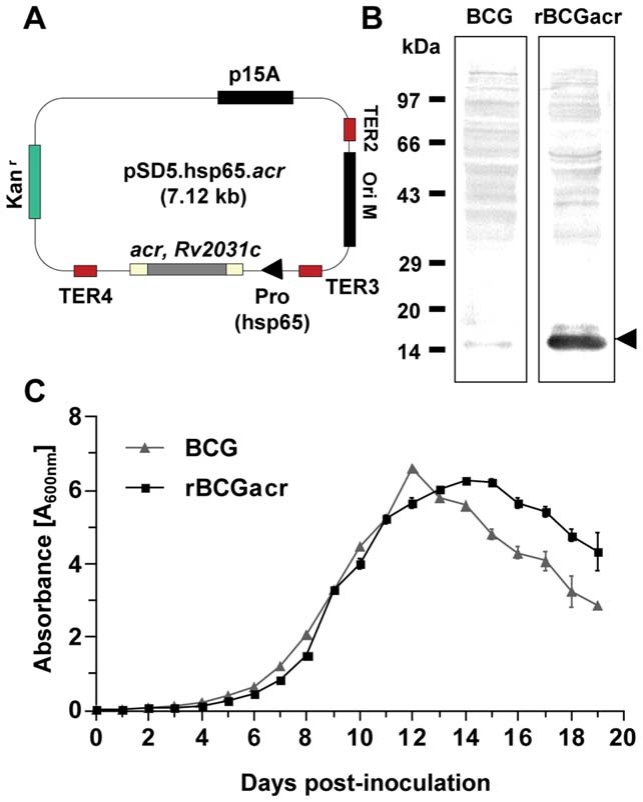
Development of rBCG strain over-expressing α-crystallin and analysis of expression and *in vitro* growth. (A) A *Mycobacteria - Escherichia coli* shuttle plasmid pSD5.hsp65.acr was engineered to over-express α-crystallin under transcriptional control of the promoter of *hsp65* gene of *M. leprae*. The recombinant plasmid was electroporated into *M. bovis* BCG to generate rBCGacr strain. (B) The whole cell lysates of BCG and rBCGacr (grown to A_600 nm_ of 0.8–1.0) were analysed for the expression of α-crystallin by SDS-PAGE and immuno-blotting by using antigen specific antibodies. By densitometric analysis, rBCGacr was found to produce a considerably higher level of 16 kDa α-crystallin protein in comparison to wild type BCG strain (∼15 fold). (C) Growth kinetics of BCG and rBCG during 19 days of culture in 7H9 medium. The A_600 nm_ of broth culture was plotted against time. Data points are presented as mean ± SE of duplicate cultures.

rBCG strain over-expressing α-crystallin of *M. tuberculosis* was prepared by transformation of BCG with a recombinant plasmid (pSD5.hsp65.acr) engineered to over-express α-crystallin under the transcriptional control of promoter of the *hsp65* gene of *M. leprae* (Fig. 1A). A comparison of the protein expression in the lysates of BCG and rBCGacr harvested from a culture of A_600 nm_ 0.8–1.0 by densitometry analysis of the immuno-blots revealed a considerably higher expression of α-crystallin in rBCG (15 fold) when compared with the parental BCG strain (Fig. 1B).

To determine whether the higher production of α-crytallin by rBCG influences its *in vitro* growth, we monitored the A_600 nm_ of BCG and rBCG cultures over a period of 19 days (Fig. 1C). Both BCG and rBCG exhibited a comparable growth pattern till late log phase (till day 11). Although, rBCG failed to achieve as high A_600 nm_ as that by wild type BCG at the end of log phase (day 12), it maintained the saturation A_600 nm_ for a considerably longer duration than the wild type BCG. In the death phase, while, BCG showed a sharp decline in A_600 nm_, the corresponding decline in the case of rBCG was considerably slower. The *in vitro* growth kinetics indicates that over-expression of α-crystallin in rBCG perhaps protects the aging bacteria to some extent against autolysis owing to the chaperonic activity of α-crystallin, which is known to stabilize the cell wall and intracellular structures. These observations are in coherence with a previous study, which showed a slower decline in the viability following the end of log-phase growth of recombinant *M. tuberculosis* over-expressing this protein [7].

### Enhanced protection conferred by α- crystallin based heterologous prime boost vaccination

**Figure 2:**
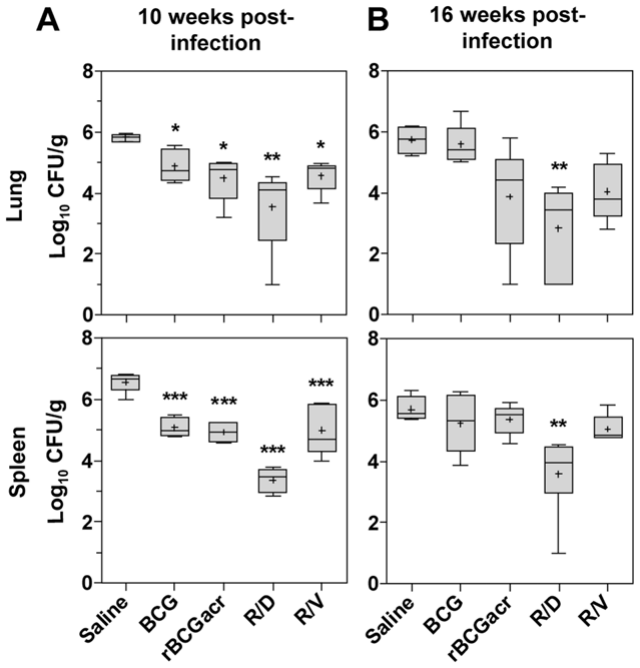
Protection by α-crystallin based prime boost regimens against *M. tuberculosis* challenge. The figure depicts the bacillary load in lungs and spleen of guinea pigs at (A) 10 weeks (n = 5) and (B) 16 weeks (n = 6) post-infection. Animals vaccinated with R/D regimen exhibited a significantly lower bacillary load in lung and spleen when compared to the unvaccinated as well as BCG vaccinated animals at both 10 weeks and 16 weeks post-infection. Log_10_ CFU is represented by box plot, wherein median values are denoted by horizontal line, the mean is represented by ‘+’, inter quartile range by boxes, and the maximum and minimum values by whiskers. R/D, rBCG prime – DNAacr boost; R/V, rBCG prime – vector boost. (*, *p*<0.05, **, *p*<0.01 and ***, *p*<0.001, when compared to the saline group, One-way ANOVA).

To evaluate the protective efficacy of α-crystallin based vaccine regimens, we first determined the influence of vaccine-induced immunity on the multiplication of *M. tuberculosis*. For this, 12 weeks after the primary immunization, guinea pigs were infected with 50–100 bacilli by aerosol route and euthanized at 10 weeks post-infection (Exp-I). As described in a seminal study by EU TB vaccine cluster, involving evaluation of 24 TB vaccine regimens in guinea pig model, the dose of infection employed in this study not only allowed induction of measurable clinical illness in 100% of control animals but also permitted discrimination with respect to BCG within a relatively short time frame (10 weeks) [8]. Under these experimental conditions, all the vaccine regimens resulted in a significantly reduced bacillary load in lungs and spleen, when compared to the unvaccinated animals (*p*<0.05) (Fig. 2A). Immunization with BCG (0.94 log_10_ and 1.48 log_10_ fewer bacilli in lungs and spleen, respectively) and rBCG (1.34 log_10_ and 1.63 log_10_ fewer bacilli in lungs and spleen, respectively) resulted in a comparable reduction in bacillary load when compared to the unvaccinated animals. However, the ‘rBCG prime-DNA boost’ regimen (R/D) conferred a far greater protection with a markedly reduced bacillary count when compared to the unvaccinated animals (2.28 log_10_ and 3.2 log_10_ fewer bacilli in lungs and spleen, respectively). Moreover, the magnitude of protection by R/D regimen was especially noteworthy, as it resulted in 1.34 log_10_ and 1.72 log_10_ fewer bacilli in lungs and spleen, respectively in comparison to BCG vaccination (*p*<0.05), (Fig. 2A).

Having established the superiority of α-crystallin based R/D regimen in restricting the bacillary multiplication in short-term experiment; we sought to evaluate the sustenance of protection by extending the post-challenge period to 16 weeks (Exp-II). As disease severity increased with time, one third of the unvaccinated animals (2/6) succumbed to the disease. Although, no mortality was observed in the BCG group, the bacillary load in the lungs and spleens of BCG vaccinated animals was observed to be as much as in the case of unvaccinated animals (surviving ones) indicating a considerable decline in the protection conferred by BCG vaccination on extending the post-challenge period to 16 weeks (Fig. 2B). In contrast, rBCG regimen still resulted in a considerably reduced bacillary load in the lungs (1.87 log_10_ fewer bacilli), but not in spleen, when compared to the unvaccinated animals. However, remarkably the R/D regimen continued to impart a significant protection as was evident from a substantially reduced bacillary load both in lungs (by 2.75 log_10_) and spleen (by 1.65 log_10_) in comparison to the BCG vaccinated animals (*p*<0.01), (Fig. 2B). The influence of boosting on bacillary reduction in case of R/D regimen was antigen specific, since no additional benefit was observed in case of rBCG prime – vector boost (R/V) regimen over rBCG vaccination.

**Figure 3:**
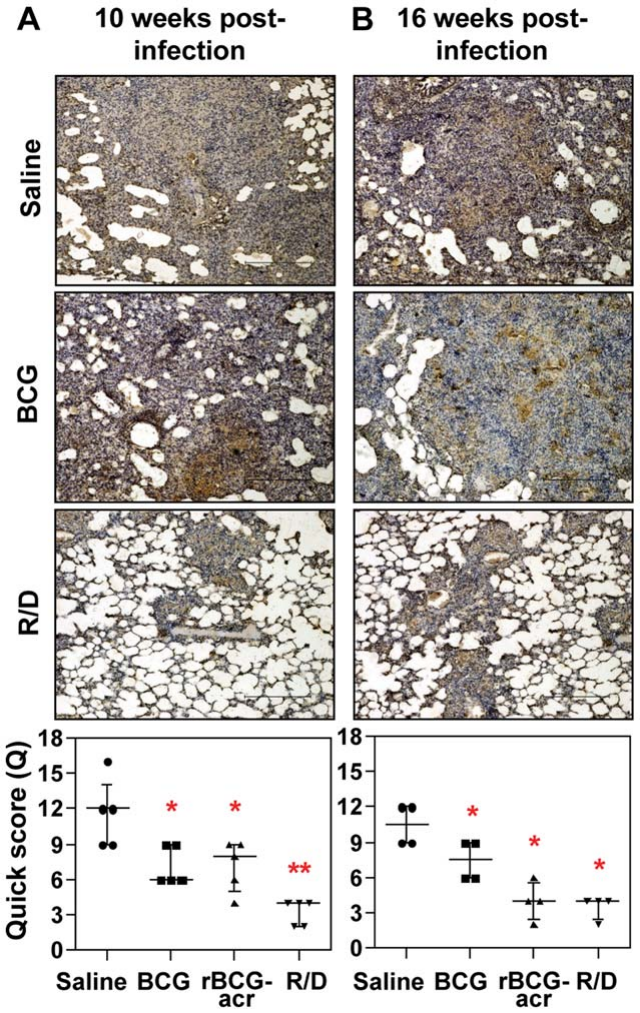
α-crystallin based prime boost vaccination reduces antigen load in pulmonary granulomas. The representative photomicrographs of lung sections show immuno-histochemical staining (brown color) for Ag85 complex proteins in pulmonary granulomas at (A) 10 weeks and (B) 16 weeks post-infection. (A) Animals in the unvaccinated group exhibited extensive staining within the granulomatous regions; BCG and rBCGacr vaccinated animals showed moderate and comparable staining; animals vaccinated with R/D regimen showed a reduced antigen staining, when compared to BCG vaccinated animals. (B) Unvaccinated animals and BCG vaccinated animals showed similar staining pattern as observed at 10 weeks; rBCGacr and R/D vaccinated animals showed a significantly reduced antigen load. Scale bar represents 1 mm. Extent (Q) of staining was measured by light microscopy [Q = intensity (I)×area (A) of staining] and represented graphically as median (± inter quartile range). R/D, rBCG prime – DNAacr boost. (*, *p*<0.05; **, *p*<0.01, when compared to the unvaccinated animals, Mann-Whitney *U* test).

In parallel, we also determined *M. tuberculosis* antigen load in lung tissues by *in situ* localization of Ag85 complex proteins that in addition to indicating the presence of live bacilli, served as a marker of dead bacilli, bacillary remnants as well as secreted antigens (a source of inflammation) [9]. We observed a strong positive correlation between antigen load and bacillary count (r = .78, *p*<0.001). Particularly, in concordance with the reduced bacillary load, animals vaccinated with R/D regimen exhibited a minimal antigen load both at 10 and 16 weeks post infection in comparison to saline and BCG immunized animals (Fig. 3). Taken together, these observations confirm that α-crystallin based R/D regimen not only exhibited a greater and sustained control on bacillary multiplication but was also efficient in antigenic clearance from the site of infection.

### Reduced gross and histopathological lesions following prime boost vaccination

**Figure 4:**
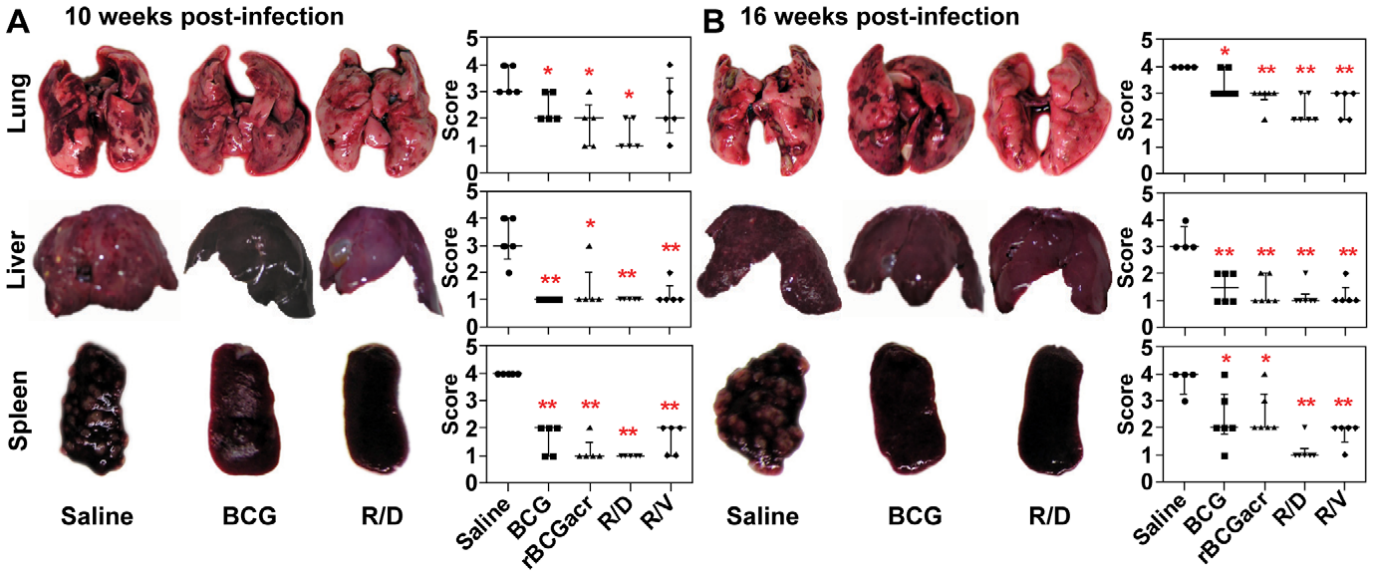
Reduction in gross pathological damage by α-crystallin based prime boost regimen. The figure depicts representative photographs and graphical depiction of gross scores of lung, liver and spleen of guinea pigs at (A) 10 weeks (n = 5) and (B) 16 weeks (n = 6) post-infection. Based on the extent of involvement, number and size of tubercles, areas of inflammation and necrosis; gross pathological scores were graded from 1–4 as described in materials and methods and represented graphically. (A) BCG, rBCGacr and R/D regimens resulted in a significant reduction in pathological lesions in lung and liver compared to the saline control; animals in R/D group also showed a significantly reduced spleen pathology, when compared to BCG vaccinated animals. (B) Animals in R/D group showed minimal pathology in all the organs, when compared to BCG vaccinated animals. Each point represents score for an individual animal and the bar depicts median (± inter quartile range) for each group. Missing data points represent the animals that succumbed to disease before euthanasia. R/D, rBCG prime – DNAacr boost; R/V, rBCG prime – vector boost. (*, *p*<0.05 and **, *p*<0.01, when compared to the saline group, Mann-Whitney *U* test).

We next examined the influence of prime boost vaccination on pathological changes in various organs of guinea pigs. At 10 weeks post-infection, unvaccinated animals exhibited severe pathology in all the organs. Immunization of animals with BCG as well as rBCG resulted in a significant decline in the number of gross lesions in the lungs and spleen compared to the unvaccinated animals. Moreover, liver of animals from both these groups showed no evident gross lesions (Fig. 4A). Noticeably, animals vaccinated with R/D regimen exhibited only a minimal pulmonary involvement with no evident gross lesions in liver as well as spleen.

**Figure 5:**
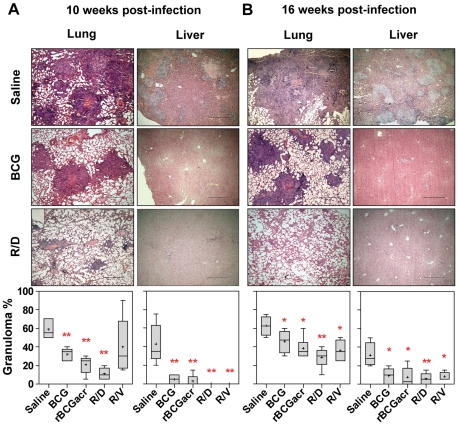
Reduced granulomatous inflammation following *M. tuberculosis* infection in animals vaccinated with R/D regimen. Representative photomicrographs of H&E stained lung and liver sections showing granulomatous pathology at (A) 10 weeks (n = 5) and (B) 16 weeks (n = 6) post-infection. Lung: at both the time points, unvaccinated animals showed the presence of coalescing granulomas with extensive necrosis; BCG and rBCG groups showed moderate involvement with well-organized discrete granulomas with or without central necrosis; animals in R/D group showed reduced granulomatous infiltration with a few diffused aggregates of inflammatory cells in the peribronchial and perivascular areas. Liver: unvaccinated animals showed moderate to high granulomatous lesions; BCG vaccinated animals showed minimal inflammatory aggregates; all the regimens based on α-crystallin showed negligible hepatic inflammation. Scale bar represents 2 mm. Granuloma % were measured by light microscopy and graphically represented by box plot (notations are described in the legend of Fig. 1). R/D, rBCG prime – DNAacr boost; R/V, rBCG prime – vector boost. (*, *p*<0.05, **, *p*<0.01, when compared to the saline group, Mann-Whitney *U* test).

Morphometric analysis of lung and liver sections further substantiated the gross pathological observations. At 10 weeks post-infection, unvaccinated animals exhibited extensive coalescing granulomas (∼60%) encompassing scattered areas of necrosis in lungs along with lymphocytic infiltration in the hepatic lobules (∼43%) (Fig. 5A). Animals vaccinated with BCG and rBCG not only exhibited reduced pulmonary consolidation (∼32% and 21%, respectively), they also showed markedly reduced hepatic inflammation (∼5%). Among the vaccinated groups, animals belonging to R/D group showed minimal pulmonary consolidation (∼11%), with the presence of only a few scattered areas of diffused infiltration, primarily in the inter-alveolar septa and peribronchial or perivascular regions, with no evidence of inflammation in liver (Fig. 5A).

The protective effect of α-crystallin based prime boost vaccination was further substantiated by pathological observations in Exp-II. While BCG and rBCG vaccinated guinea pigs exhibited an increased disease severity with time, animals vaccinated with R/D regimen exhibited minimal gross lesions in liver and spleen along with a considerably lower pulmonary pathology compared to BCG and rBCG vaccinated animals (Fig. 4B). Histological analysis also supported the gross pathological findings (Fig. 5B).

**Figure 6:**
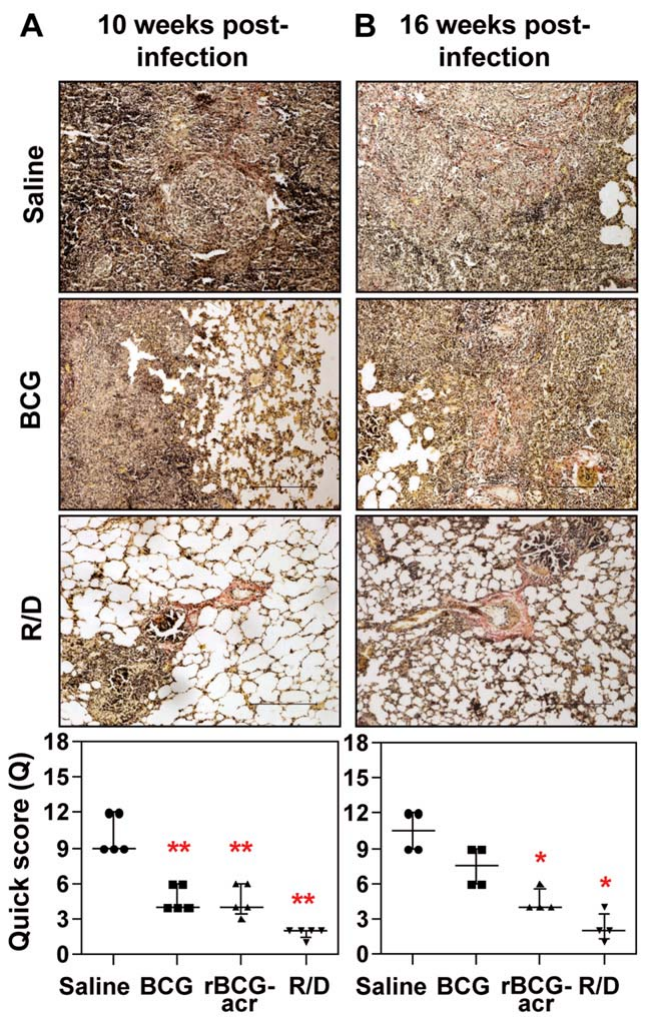
Influence of α-crystallin based prime boost vaccination on pulmonary fibrosis following *M. tuberculosis* infection. The figure depicts representative photomicrographs of Van Gieson stained lung sections of guinea pigs euthanized at (A) 10 weeks and (B) 16 weeks post-infection. (A) Unvaccinated animals showed extensive fibrosis characterized by widespread presence of thick bands of collagen fibers (red color) in the granulomatous areas; BCG and rBCGacr vaccinated animals showed a moderate staining with the presence of thin bands of collagen. Animals vaccinated with R/D regimen showed no evident signs of collagen staining other than the usual occurrence of collagen in the peri-bronchial and peri-vascular areas. (B) Unvaccinated animals and BCG vaccinated animals showed similar staining pattern as observed at 10 weeks; rBCGacr and R/D vaccinated animals showed negligible staining. Scale bar represents 1 mm. Extent (Q) of pulmonary fibrosis was measured by light microscopy [Q = Intensity (I)×area (A) of staining] and represented graphically as median (± inter quartile range). R/D, rBCG prime – DNAacr boost. (*, *p*<0.05; **, *p*<0.01, when compared to the unvaccinated animals, Mann-Whitney *U* test).

Further, while, unvaccinated animals exhibited obliteration of alveolar structure due to widespread collagen deposition in the granulomatous areas in the lungs, no evident signs of collagen deposition were observed in the lungs of animals vaccinated with R/D regimen at both 10 and 16 weeks post-infection (Fig. 6). Collagen deposition in BCG and rBCG vaccinated animals was although significantly reduced when compared to the unvaccinated animals (*p*<0.05), it remained markedly higher in comparison to R/D vaccinated animals (Fig. 6). These observations suggest that vaccination with R/D regimen, in addition to reducing the bacillary burden, imparts a marked protection against collateral pathological damage.

### Vaccination induced cytokine response and its influence on immunopathology and protection

**Figure 7:**
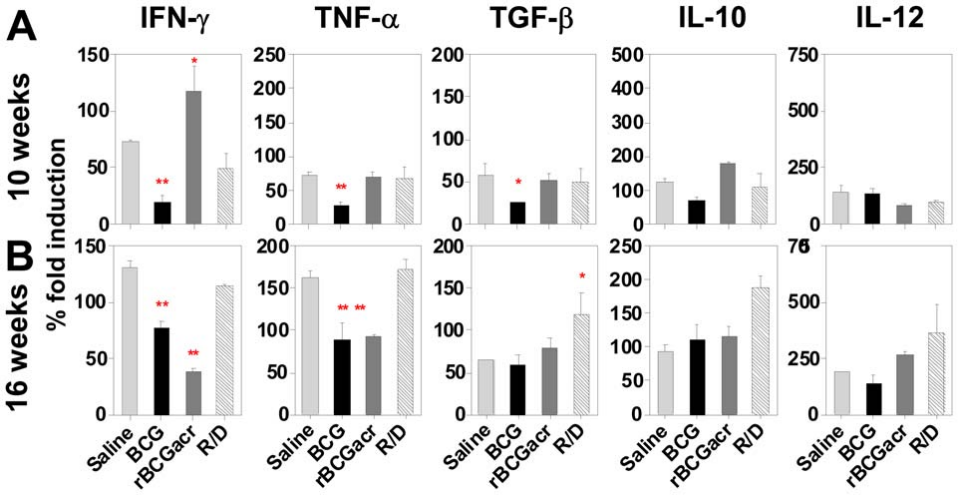
α-crystallin based prime boost vaccination induces dynamic changes in the cytokine milieu in lungs. Expression of various cytokines was measured in the lung tissues of vaccinated and saline treated guinea pigs at (A) 10 weeks and (B) 16 weeks post-infection by semi-quantitative real time RT-PCR by using gene specific primers. The data were normalized to 18S rRNA levels and then normalized to the values of uninfected animals to obtain ΔΔCt values. The % fold induction values were measured [2^−ΔΔCt^×100] and were graphically represented as mean (± SE). For cytokine measurement, 3 lung samples were chosen randomly from each group. R/D, rBCG prime – DNAacr boost. (*, *p*<0.05 and **, *p*<0.01, when compared to the saline group, One-way ANOVA).

Considering the observed differences in the levels of protection imparted by various α-crystallin based vaccine regimens, we next attempted to delineate the immunological responses that differentiate a successful vaccine regimen from the one that fails to provide consistent protection over a prolonged period of time. To address this issue, profiling of various pro and anti-inflammatory cytokines was carried out by real time RT-PCR of the mRNA isolated from the lungs of guinea pigs at 10 and 16 weeks post-infection. At 10 weeks post-infection BCG vaccinated animals exhibited very low levels of all the cytokines studied (IFNγ, TNFα, TGFβ, IL10 and IL12) (Fig. 7A). At this time point, rBCG vaccinated animals exhibited relatively higher levels of IFNγ, TNFα, TGFβ and IL10 compared to BCG vaccinated animals. However, the level of IL12 in rBCG-vaccinated animals remained relatively lower compared to the BCG vaccinated animals. Similarly, animals vaccinated with R/D prime boost regimen also exhibited relatively higher levels of IFNγ, TNFα and TGFβ compared to BCG vaccinated animals along with a relatively lower level of IL12 (Fig. 7A).

At 16 weeks post-infection, unvaccinated animals exhibited a marked up-regulation in the levels of IFNγ and TNFα in comparison to 10 weeks post-infection. Similarly, BCG immunized animals exhibited a significant up-regulation in the levels of inflammatory cytokines (IFNγ and TNFα) (*p*<0.05), when compared to 10 weeks time point (Fig. 7B). However, the levels of IL10, IL12 and TGFβ in these groups remained unaltered with time. Contrary to BCG, on extending the time of evaluation to 16 weeks post-infection, rBCG vaccination resulted in a considerable decline in the levels of IFNγ and IL10; a significant increment in the levels of IL12 (*p*<0.05), while the levels of TNFα and TGFβ remained unaltered. Notably, animals vaccinated with R/D regimen exhibited a marked up-regulation in the expression of all the cytokines investigated, with the highest up-regulation noticed in case of IL12, when compared to the 10 weeks time point (Fig. 7B).

Thus, R/D regimen, which imparted the highest protection amongst all the vaccine regimens, exhibited the most striking pattern of cytokine expression. The animals from this group not only exhibited a significant up-regulation of pro-inflammatory cytokines but the levels of anti-inflammatory cytokines were also elevated in comparison to BCG and rBCG vaccinated animals (Fig. 7B). While, we did not observe any correlation between an increase in the level of IFNγ alone with the degree of protection, it was evident that simultaneous up-regulation of IL12 as well as anti-inflammatory cytokines like TGFβ and IL10 is crucial for a sustained protection as observed in the case of R/D regimen. Our data, thus suggests that to understand the role of cytokines in protective immunity and to correlate the cytokine milieu with the vaccine induced protection, it may be prudent to take the dynamic interplay of various cytokines into consideration instead of measuring a single cytokine, such as IFNγ.

### Induction of multi-functional CD4 Th1 cell response by R/D regimen confers enhanced protection in murine model

Although, dynamic changes in the pulmonary cytokine milieu provided crucial information regarding the involvement of these cytokines in protection against TB, non-availability of immunological reagents in guinea pigs limited our ability to study the T cell effector functions and define the immuno-correlates of protection in guinea pigs. Thus, we next investigated the immune responses induced by various vaccine regimens along with the evaluation of their protective efficacy in murine model.

Murine model has been extensively employed to understand the role of Th1 responses in *M. tuberculosis* infection and vaccine-induced protection [10], [11]. While, generation of effector cytokines, such as IFNγ and TNFα by the Th1 cells are known to be crucial for the resolution of *M. tuberculosis* infection, IL2 production by these T helper cells aids in the maintenance of memory immunity against infection [12], [13]. Based on this, we carried out a functional analysis of cytokine producing CD4 T cells by multi-parameter flow cytometry. We assessed the Th1 cell response by staining the spleen cells for CD4 T cell marker along with IFNγ, TNFα, and IL2 after stimulation with PPD and α-crystallin. Based on the expression of various combinations of these cytokines, CD4 T cells were divided in to 7 distinct populations as described in [14], which define the quality of immune response. The immune responses were compared among BCG, rBCG and R/D groups at 12 weeks after the primary immunization (at the time of infection) with respect to the sham immunized animals.

**Figure 8:**
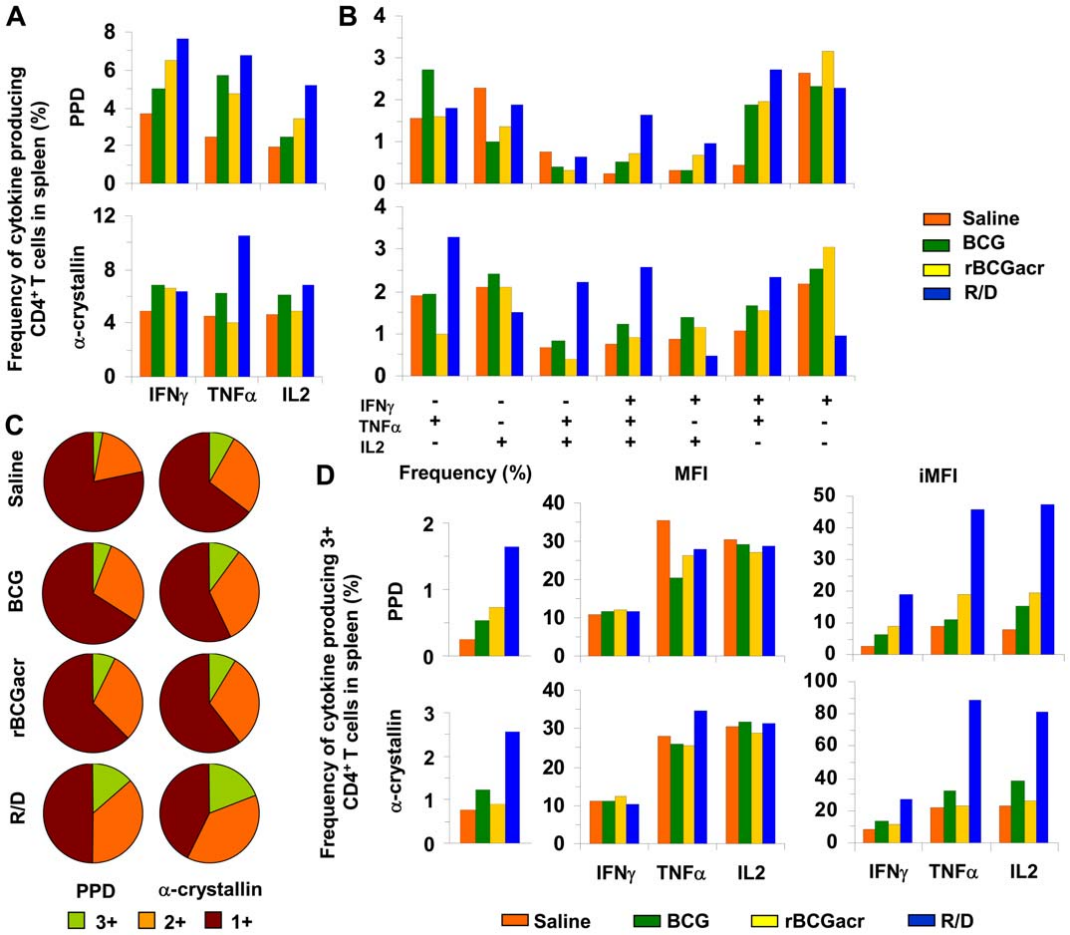
Induction of CD4 Th1 cell responses by R/D immunization. At 12 weeks post-immunization, T lymphocytes were purified from PPD and α-crystallin stimulated splenocytes (pooled from four mice per group) and stained for cell surface marker (CD4) along with intracellular staining for IFNγ, TNFα and IL2 followed by FACS analysis. Frequency of IFNγ, TNFα and IL2 producing cells was determined on CD4 T cell gated population. (A) Total frequency (%) of PPD and α-crystallin specific cytokine producing CD4 T cells in spleen. (B) Frequency (%) of PPD and α-crystallin specific CD4 T cells expressing each of the seven combinations of IFNγ, TNFα and IL2. (C) Proportion of PPD and α-crystallin specific CD4 T cells producing one, two or three cytokines. (D) Frequency (%) of PPD and α-crystallin specific 3^+^ CD4 T cells in spleen along with MFI and iMFI for IFNγ, TNFα and IL2. R/D, rBCG prime – DNAacr boost.

At 12 weeks post-immunization, although a comparable frequency of CD4 and CD8 T cells was observed in splenocytes isolated from all the groups (data not shown), a marked functional heterogeneity was observed in the CD4 T cell subsets isolated from different groups in terms of their ability to produce IFNγ, TNFα and IL2 (Fig. 8). R/D vaccination resulted in a very high total frequency of PPD specific IFNγ^+^, TNFα^+^ or IL2^+^ CD4 T cells when compared with BCG, rBCG as well as sham immunized animals (Fig. 8A). In addition, the total frequency of α-crystallin specific TNFα producing CD4 T cells was also considerably higher in R/D group (Fig. 8A).

A more comprehensive functional analysis of CD4 T cells producing various combinations of cytokines revealed the presence of a markedly higher frequency of PPD as well as α-crystallin specific 3^+^ (IFNγ^+^TNFα^+^ IL2^+^) and 2^+^ (producing any two cytokines) CD4 T cells in R/D vaccinated animals compared to BCG, rBCG and sham immunized animals (Fig. 8B and 8C). Among the 2^+^ CD4 T cells, a considerably higher frequency of α-crystallin specific TNFα^+^IL2^+^ and IFNγ^+^TNFα^+^ cells were observed in case of R/D vaccinated animals (Fig. 8B). In addition, R/D vaccinated animals also exhibited a relatively higher frequency of PPD specific IFNγ^+^TNFα^+^ CD4 T cells. On comparing different vaccinated groups, although, we did not notice any considerable difference in the median fluorescence intensities (MFIs) of 3^+^ CD4 T cells, integrated MFI (iMFI = %frequency×MFI) for all the three cytokines of these cells remained markedly higher in R/D vaccinated animals compared to rest of the groups (Fig. 8D). We also looked for a potential role of CD8 T cells following a similar analysis, however, we did not observe any characteristic association of vaccine induced protection with these cells in this study (data not shown).

**Figure 9:**
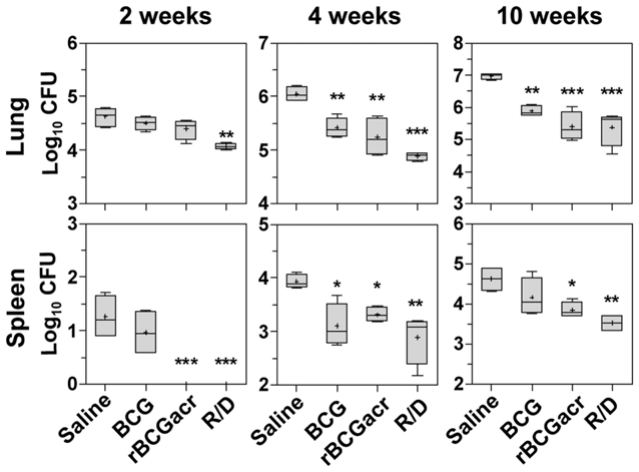
α-crystallin based vaccination provides protection against *M. tuberculosis* challenge in mice. Mice were infected 12 weeks after primary immunization and euthanized at 2, 4 and 10 weeks post-infection and lung and spleen bacillary load were determined. Log_10_ CFU is graphically represented by box plot (notations are described in the legend of Fig. 1). Animals vaccinated with R/D regimen exhibited a significantly lower bacillary load in lung and spleen when compared to the unvaccinated as well as BCG vaccinated animals at 2, 4 and 10 weeks post-infection. R/D, rBCG prime – DNAacr boost. (*, *p*<0.05, **, *p*<0.01 and ***, *p*<0.001, when compared to the saline group, One-way ANOVA).

Next, we evaluated the protective efficacy of various vaccine regimens in mice at 2, 4 and 10 weeks post-infection. On comparing the protective efficacy of these vaccine regimens at 2 weeks post infection, rBCG vaccination did not result in any significant reduction in bacillary load in the lungs when compared to the sham immunized animals, although, it slowed down the dissemination of bacilli to spleen (Fig. 9). However, the R/D regimen exhibited the highest protection among all the vaccinated groups (Fig. 9). In addition to significantly reducing the bacillary load in lungs compared to the unvaccinated animals (0.55 log_10_ fewer bacilli, *p<*0.01), this regimen also slowed down the dissemination of bacilli to spleen to the extent that no bacillary growth was detected in spleen at 2 weeks post-infection. These observations are especially noteworthy, since, at such an early time point, BCG vaccination, as seen in this study (Fig. 9) and in another seminal study [15] does not begin to exhibit any significant control on bacillary multiplication in lung or spleen in comparison to the sham immunization.

By 4 weeks post-infection, all the vaccine regimens resulted in a marked reduction in lung and spleen bacillary load when compared to sham immunization (Fig. 9). However, R/D regimen conferred the highest protection with a markedly reduced bacillary count when compared to the unvaccinated animals [1.16 log_10_ and 1.05 log_10_ fewer bacilli in lungs (*p*<0.001) and spleen (*p*<0.01), respectively]. Moreover, the magnitude of protection in lungs by R/D regimen was especially noteworthy, as it resulted in 0.52 log_10_ fewer bacilli in lungs in comparison to BCG vaccination (*p*<0.05). At this time point, rBCG vaccination however conferred a comparable protection to that observed in case of BCG vaccination.

Further, at 10 weeks post-infection, R/D regimen continued to impart the highest protection, as was evident from a markedly reduced bacillary load both in lungs and spleen in comparison to the unvaccinated animals [1.6 log_10_ and 1.1 log_10_ fewer bacilli in lungs (*p*<0.001) and spleen (*p*<0.01), respectively] (Fig. 9). Moreover, the magnitude of protection conferred by R/D regimen remained significantly higher in comparison to BCG vaccination at 10 weeks post-infection (with 0.5 log_10_ and 0.65 log_10_ fewer bacilli in both lung and spleen, *p*<0.05), (Fig. 9). At this time point, although, BCG as well as rBCG vaccination resulted in a significant reduction in lung bacillary load when compared to sham immunization [1.1 log_10_ (*p*<0.01) and 1.57 log_10_ (*p*<0.001) fewer bacilli, respectively], the reduction in splenic bacillary load was statistically significant only in case of rBCG group (0.77 log_10_, *p*<0.05).

Altogether, the observations in the murine model indicate that the induction of a multifunctional Th1 cell response by R/D regimen significantly contributes to the enhanced protection against *M. tuberculosis* infection.

## Discussion

Latency associated antigens have been proposed to be prospective candidates for vaccine development against TB, however, only a few studies to date have explored the vaccine potential of such antigens [16], [17], [18], [19]. In this study, we evaluated the protective efficacy of a heterologous prime boost approach targeting α-crystallin - a key latency-associated antigen against TB. We show that the prime boost approach based on α-crystallin provides a superior and extended protection against *M. tuberculosis* infection in guinea pigs. The pathology and cytokine expression data from guinea pigs support the protective potential of α-crystallin based prime boost approach. Further, the findings in murine model provide evidence for the importance of multi-functional CD4 Th1 response in protection against *M. tuberculosis* infection. Vaccination with R/D regimen not only induces a heightened multi-functional CD4 Th1 cell response, but also demonstrates a significant control on the bacillary multiplication in the early as well late phase of infection.

Two main caveats of BCG vaccine are related to its variable efficacy and inability to impart sterilizing immunity against *M. tuberculosis* infection resulting in a large pool of latently infected individuals. In a recent study, Henao-Tamayo and colleagues reported that generation of inadequate central memory T cell response by BCG vaccine renders the host susceptible to TB infection or reactivation at the onset of adulthood [20]. In addition, Vekemen *et al.* indicated that inability of BCG to provide sterilizing immunity against primary *M. tuberculosis* infection may also stem from the inadequate immune response that it generates against latency associated antigens, such as α-crystallin [5]. Since, a majority of active TB cases emerge from the reactivation of latent infections, the need for targeting the latency-associated antigens for the development of new vaccines against TB cannot be over emphasized [1]. Here, we demonstrate that enriching BCG with latency antigen α-crystallin and subsequently boosting the immunity in an antigen specific manner not only results in an efficient CD4 Th1 response but also imparts a significantly superior protection over the conventional BCG vaccination.

Vaccination induced alterations in the cytokine milieu dictate the outcome of the infection [21]. In this study, for the first time, a new TB vaccine regimen has been evaluated to analyze the association between five prominent cytokines and vaccine induced protection in guinea pigs, to provide an understanding about how the dynamics of pulmonary cytokine profile influences the fate of an infection. While, the levels of IFNγ or TNFα alone did not provide any apparent association with the degree of protection conferred by different vaccine regimens, changes in the levels of other pro- and anti-inflammatory cytokines, such as IL12, IL10 and TGFβ provided a distinct association of these cytokines with the observed protection. A notable observation in the unvaccinated and BCG vaccinated animals was the dysregulation of TNFα and IFNγ expression with time, resulting in an exacerbation of pathology as was evident from an increased granulomatous inflammation and necrosis in the lungs. However, the expression of immuno-regulatory or anti-inflammatory cytokines like IL10 and TGFβ remained unaltered in these groups. In contrast, the animals vaccinated with α-crystallin based R/D regimen, although exhibited similar up-regulation of TNFα and IFNγ, their influence was countered stringently by a commensurate up-regulation of anti-inflammatory cytokines (IL10 and TGFβ), thereby preventing collateral pathological damage. In addition to these cytokines, IL12 also exhibited a close association with the degree of protection. Thus, these observations suggest that an increase in the levels of IL12 as well as anti-inflammatory cytokines, such as TGFβ and IL10 from 10 weeks to 16 weeks post-infection are perhaps crucial for the extended protection and a concomitantly reduced pathology observed in the case of R/D regimen. These observations support a previous study that has shown a significant reduction in bacillary load and increased survival of animals on administration of IL12 following *M. tuberculosis* infection [22]. The enhancement in the levels of anti-inflammatory cytokines like IL10 and TGFβ has also been reported to be essential for the resolution of granulomatous inflammation making them crucial for alleviation of disease symptoms (reviewed in [23]). Our findings are also in coherence with previous studies, which have demonstrated that if the production of TNFα is not counteracted by anti-inflammatory cytokines, it results in massive lung destruction and ultimately death as observed in the case of unvaccinated animals [10], [21], [24], [25], [26]. A seminal study by Ly *et al* [21] demonstrated that one of the key elements in the protection of guinea pigs by BCG vaccination relates to production of IFNγ along with TNFα post-infection in comparison to the unvaccinated animals, wherein, excessive quantities of TNFα are produced in the absence of IFNγ resulting in an inefficient control of bacillary growth accompanied by excessive pathology. However, once, BCG vaccinated animals control the bacillary multiplication, prominence of TGFβ mRNA was observed at the lesion sites, which was accompanied by resolution of pathology [21]. Although, these associations in our study were deduced from the measurement of mRNA levels and not by the direct measurement of cytokines at protein level, they do suggest potential involvement of these cytokines in mediating protection against TB. Moreover, several studies have employed real time RT-PCR for cytokine measurement in case of guinea pigs for which immunological reagents are still unavailable [21], [27]. These findings in guinea pig model advocate that the measurement of a battery of cytokines, rather than a single cytokine, such as IFNγ is more likely to provide an appropriate association with vaccine-induced protection.

Recent studies have emphasized that diseases, such as tuberculosis, leishmaniasis and HIV/AIDS, which particularly require Th1 response for an efficient control, rely considerably on the presence of multi-functional memory and effector T cells [28], [29], [30]. Our observations in murine model further indicate potential involvement of multifunctional (3^+^) CD4 Th1 cell response in conferring protection against *M. tuberculosis* infection by R/D vaccination. Moreover, induction of a larger repertoire of both TNFα^+^IL2^+^ and IFNγ^+^TNFα^+^ 2^+^ CD4 T cells by R/D regimen perhaps provides a reservoir of long-term central memory CD4 T cells as well as a pool of effector cells. In contrast, BCG vaccination, which results in a relatively lower proportion of 3^+^ and 2^+^ cytokine producing CD4 T cells, is limited in its ability to mediate an efficient and sustained protection. Although, the data from mice study, wherein we analysed the immunological responses on pooled samples need to be interpreted cautiously, it does indicate a crucial role of multifunctional CD4 T cell response against *M. tuberculosis* infection. Moreover, these findings are in coherence with a seminal study, which indicated that it is the ability of Th1 cells to produce multiple cytokines, which determines the potency of these cells in mediating protection against intracellular infections, such as *Leishmania major* and *M. tuberculosis* [29]. Although, caution should be exercised to extrapolate the mouse immunogenicity data to guinea pigs due to inherently different response of the two species to *M. tuberculosis* infection and a formal demonstration of the involvement of multifunctional T cells in mediating protection in guinea pigs is still awaited due to the unavailability of immunological regents for guinea pigs, the immunogenicity and protective efficacy studies in murine model do indicate a crucial role of multifunctional Th1 cell response in mediating protection against TB.

The ‘rBCG prime - DNA boost’ regimen employed in this study provides multiple advantages and possibilities in terms of its clinical relevance. Firstly, in this regimen, the use of rBCG in place of BCG in newborn children will not only preserve the valuable attributes of BCG, but will also result in an efficient immune response and superior protection against pulmonary TB. Secondly, a booster dose of DNA vaccine would further enhance and sustain the rBCG-induced immunity. In a significant finding, Pathan *et al.* reported that the immunomodulatory effect of an efficient booster vaccine remains unaltered irrespective of the time span between the primary BCG vaccination and boosting as these investigators found no significant difference in the magnitude of immune responses generated, when the booster was administered a few months or many years after BCG vaccination [31]. Hence, the advantage of boosting with DNAacr witnessed in this study can be exploited at any appropriate age. Thirdly, induction of α-crystallin specific memory immunity will aid in the enhanced recognition and clearance of latent bacilli. Hence, vaccination with ‘rBCG prime - DNA boost’ regimen based on α-crystallin is likely to reduce the incidence of latent and reactivation TB. While, further studies would be required to demonstrate these plausible advantages, a successful demonstration of α-crystallin based heterologous prime boost vaccination approach in guinea pig as well as mouse model definitely provides a paradigm to explore the potential of latency antigens for developing prophylactic and therapeutic vaccines against TB.

## Materials and Methods

### Ethics statement

Guinea pig experiments were reviewed and approved by the Institutional Animal Ethics Committee of National JALMA Institute for Leprosy & Other Mycobacterial Diseases, Agra, India (Permit number: 101/1997/CPCSEA). Mice experiments were reviewed and approved by the Institutional Animal Ethics Committee of University of Delhi South Campus, New Delhi, India (Permit number: 159/1999/CPCSEA). All animals were routinely cared for according to the guidelines of CPCSEA (Committee for the Purpose of Control and Supervision on Experiments on Animals), India.

### Bacterial strains, animals and preparation of antigens for immunization

For preparation of rBCGacr, a *Mycobacteria - Escherichia coli* shuttle plasmid pSD5.pro [32], [33] was engineered to over-express α-crystallin gene (*Rv2031c*) under the transcriptional control of the promoter of *hsp65* gene of *M. leprae*. Briefly, the gene encoding α-crystallin (*Rv2031c*) was PCR amplified by using *M. tuberculosis* H37Rv genomic DNA as the template and gene specific primers (forward- 5′gggcatcatatggccaccaccc 3′ and reverse- 5′gggacgcgtcagttggtggaccggatgtg 3′). The PCR amplicon was then cloned into pSD5.hsp65 at *Nde* I and *Mlu* I restriction sites and the resulting construct was designated as pSD5.hsp65.acr. The recombinant plasmid was electroporated into wild type *M. bovis* BCG and selected on MB7H11 plates containing Kanamycin (25 µg/ml).

*M. tuberculosis* (H37Rv strain), *M. bovis* BCG (Danish strain) and rBCG cells were grown to mid-log phase in Middle Brook 7H9 media and stocks were prepared as described [9]. DNA vaccine, pAK4-αcry - expressing α-crystallin was prepared as described [6]. Outbred guinea pigs (Dunkin Hartley strain, female, 200–300 g) were procured from Disease Free Small Animal House Facility, HAU, India and maintained in the BSLIII facility at National JALMA Institute for Leprosy & Other Mycobacterial Diseases, Agra, India. Mice (Balb/c strain, female, 6–8 weeks) were procured from National Centre for Laboratory Animal Science, Hyderabad, India and maintained in the BSLIII facility at University of Delhi South Campus, New Delhi, India.

### *In vitro* growth of BCG and rBCG

BCG and rBCG were grown in MB7H9 broth supplemented with 1× ADC and 0.2% Tween 80. Growth was assessed at the intervals of 24 hr for 19 days by measuring the absorbance of the culture spectro-photometrically at a wavelength of 600 nm (A_600 nm_).

### Immunization of guinea pigs and evaluation of protective efficacy against *M. tuberculosis* infection

For evaluation of protective efficacy two guinea pig experiments were carried out. Guinea pigs in groups of 5–6 were immunized with the following regimens: (i, ii) 5×10^5^ CFU of either BCG or rBCG in 100 µl of saline by i.d. route, (iii, iv) rBCG once, followed by a booster dose of DNA vaccine or vector (100 µg in 100 µl of saline) by i.m. route at 6 weeks (R/D and R/V) and (v) 100 µl of saline by i.d. route (control group). Guinea pigs were infected 12 weeks after the primary immunization with ∼50–100 bacilli of virulent *M. tuberculosis* H37Rv *via* the respiratory route in an aerosol chamber (Glasscol Inc.).

Animals were monitored regularly for change in body weight and general body condition as an indicator of disease progression and were euthanized at 10 weeks (Exp-I, n = 5) and 16 weeks (Exp-II, n = 6) post-infection by intraperitoneal injection of Thiopentone sodium (100 mg/kg body weight) (Neon Laboratories Ltd.). After dissecting the animals, lung, liver and spleen were examined for gross pathological changes and scored by using the Mitchison scoring system [34] with minor modifications as described previously [9]. For histopathological evaluation, three lung lobes (right caudal, middle and cranial) and a portion of left dorsal lobe of liver were removed and fixed in 10% buffered formalin. Left caudal lung lobe and cranial portion of spleen were aseptically removed for the measurement of bacillary load. Lung and spleen bacillary load were measured along with the evaluation of histopathological changes as described [9]. The detection limit in case of both lung and spleen CFU was 1.0 log_10_ CFU/g. For isolation of RNA to be used for real time RT-PCR studies, a portion of left cranial lung lobe was stored in RNA later (Ambion) at −20°C.

### Immunohistochemistry and image analysis

For *in situ* localization of *M. tuberculosis* antigens in the sections of guinea pig lungs, immunohistochemical (IHC) staining was carried out and analysed for area and intensity of staining as described earlier [9].

### RNA isolation and real time RT-PCR

Real time PCR was performed by using SYBR green PCR Master Mix (Applied Biosystems) by using the cDNA synthesized from total RNA isolated from the lungs of guinea pigs as described [9]. Sequences of the primers for guinea pig IFNγ, TNFα, TGFβ, IL10, IL12 and 18S rRNA were as described [9], [35].

### Evaluation of protective efficacy and immune responses in mice

To further confirm the protective efficacy observed in guinea pig experiments, we carried out a similar protection study in mouse model. Mice in groups of 4 were immunized and infected following the same protocol as used for guinea pigs. At 2, 4 and 10 weeks post-infection, bacillary load in lungs and spleen was determined. Immune responses were evaluated at 12 weeks post-immunization (at the time of infection).

### Cell Isolation, stimulation and flow cytometry

Spleen cells were isolated as described [13] and cultured in RPMI-GlutaMAX™ (containing 10% heat inactivated FBS and 1× antibiotic-antimycotic) (Invitrogen). Cells were then stimulated with PPD (20 µg/ml) (Statens Serum Institut) or purified α-crystallin (40 µg/ml) in RPMI for 2 hr at 37°C. GolgiStop was added according to the manufacturer's instruction and cells were incubated for an additional 4 hr before intracellular cytokine staining.

Following stimulation, cells were washed with PBS containing 2% heat inactivated FBS and T lymphocytes were purified by using BD IMag™ Mouse T Lymphocyte Enrichment Kit according to the manufacturer's instructions. Briefly, a cocktail of biotinylated antibodies were added to the cells that simultaneously bind erythrocytes and leukocytes but not T lymphocytes. The cells were then washed to remove excess antibodies followed by the addition of BD IMag™ Streptavidin. Unlabelled T lymphocytes were then enriched by negative selection by placing them within the magnetic field of the BD IMagnet™ as per the manufacturer's instructions. Cells were washed and incubated with BD Fc Block™ (CD16/CD32 mAb to block Fc binding). Subsequently, the cells were stained for cell surface markers, CD4 (FITC) or CD8 (FITC) along with intracellular cytokines, IFNγ (RPE), TNFα (PerCPCy5.5) and IL-2 (APC) by using the BD Cytofix/Cytoperm kit according to the manufacturer's instructions. Cells were washed twice with Cytoperm and finally resuspended in sheath fluid. Cells (50,000–100,000) were acquired by using a FACS Calibur flow-cytometer (by using Cell Quest Pro software) and analyzed by using FlowJo software (Tree Star). Cell frequency, median fluorescence intensity (MFI) and integrated MFI (iMFI = % frequency×MFI) for different cytokines were calculated. For cell stimulation and FACS analysis, cells pooled from all 4 mice from a group were used. All the reagents for cell isolation and antibodies for FACS staining were obtained from BD Pharmingen, except where mentioned otherwise.

### Statistical analysis

For the comparison of CFU and % fold induction in mRNA expression level, One-way ANOVA was carried out followed by Tukey Test. Non-parametric Mann-Whitney *U* test were employed for the comparison of gross pathological score, granuloma % and IHC score. Differences were considered significant, when *p*<0.05. For statistical analysis SPSS (Version. 10.0, SPSS Inc.) and Prism 5 software (Version 5.01; GraphPad Software Inc.) were used.

## References

[1] Smith PGaMAR (1994). Epidemiology of tuberculosis.

[2] Colditz GA, Brewer TF, Berkey CS, Wilson ME, Burdick E (1994). Efficacy of BCG vaccine in the prevention of tuberculosis. Meta-analysis of the published literature.. JAMA.

[3] Sherman DR, Voskuil M, Schnappinger D, Liao R, Harrell MI (2001). Regulation of the Mycobacterium tuberculosis hypoxic response gene encoding alpha -crystallin.. Proc Natl Acad Sci U S A.

[4] Cunningham AF, Spreadbury CL (1998). Mycobacterial stationary phase induced by low oxygen tension: cell wall thickening and localization of the 16-kilodalton alpha-crystallin homolog.. J Bacteriol.

[5] Vekemans J, Ota MO, Sillah J, Fielding K, Alderson MR (2004). Immune responses to mycobacterial antigens in the Gambian population: implications for vaccines and immunodiagnostic test design.. Infect Immun.

[6] Khera A, Singh R, Shakila H, Rao V, Dhar N (2005). Elicitation of efficient, protective immune responses by using DNA vaccines against tuberculosis.. Vaccine.

[7] Yuan Y, Crane DD, Barry CE (1996). Stationary phase-associated protein expression in Mycobacterium tuberculosis: function of the mycobacterial alpha-crystallin homolog.. J Bacteriol.

[8] Williams A, Hatch GJ, Clark SO, Gooch KE, Hatch KA (2005). Evaluation of vaccines in the EU TB Vaccine Cluster using a guinea pig aerosol infection model of tuberculosis.. Tuberculosis (Edinb).

[9] Jain R, Dey B, Dhar N, Rao V, Singh R (2008). Enhanced and enduring protection against tuberculosis by recombinant BCG-Ag85C and its association with modulation of cytokine profile in lung.. PLoS ONE.

[10] Flynn JL, Chan J (2001). Immunology of tuberculosis.. Annu Rev Immunol.

[11] Kaufmann SH (2005). Recent findings in immunology give tuberculosis vaccines a new boost.. Trends Immunol.

[12] Lindenstrom T, Agger EM, Korsholm KS, Darrah PA, Aagaard C (2009). Tuberculosis subunit vaccination provides long-term protective immunity characterized by multifunctional CD4 memory T cells.. J Immunol.

[13] Forbes EK, Sander C, Ronan EO, McShane H, Hill AV (2008). Multifunctional, high-level cytokine-producing Th1 cells in the lung, but not spleen, correlate with protection against Mycobacterium tuberculosis aerosol challenge in mice.. J Immunol.

[14] Seder RA, Darrah PA, Roederer M (2008). T-cell quality in memory and protection: implications for vaccine design.. Nat Rev Immunol.

[15] Smith DW, McMurray DN, Wiegeshaus EH, Grover AA, Harding GE (1970). Host-parasite relationships in experimental airborne tuberculosis. IV. Early events in the course of infection in vaccinated and nonvaccinated guinea pigs.. Am Rev Respir Dis.

[16] Schuck SD, Mueller H, Kunitz F, Neher A, Hoffmann H (2009). Identification of T-cell antigens specific for latent mycobacterium tuberculosis infection.. PLoS One.

[17] Roupie V, Romano M, Zhang L, Korf H, Lin MY (2007). Immunogenicity of eight dormancy regulon-encoded proteins of Mycobacterium tuberculosis in DNA-vaccinated and tuberculosis-infected mice.. Infect Immun.

[18] Wang LM, Shi CH, Fan XL, Xue Y, Bai YL (2007). Expression and immunogenicity of recombinant Mycobacterium bovis Bacillus Calmette-Guerin strains secreting the antigen ESAT-6 from Mycobacterium tuberculosis in mice.. Chin Med J (Engl).

[19] Aagaard C, Hoang T, Dietrich J, Cardona PJ, Izzo A A multistage tuberculosis vaccine that confers efficient protection before and after exposure.. Nat Med.

[20] Henao-Tamayo MI, Ordway DJ, Irwin SM, Shang S, Shanley C (2010). Phenotypic definition of effector and memory T-lymphocyte subsets in mice chronically infected with Mycobacterium tuberculosis.. Clin Vaccine Immunol.

[21] Ly LH, Russell MI, McMurray DN (2007). Microdissection of the cytokine milieu of pulmonary granulomas from tuberculous guinea pigs.. Cell Microbiol.

[22] Flynn JL, Goldstein MM, Triebold KJ, Sypek J, Wolf S (1995). IL-12 increases resistance of BALB/c mice to Mycobacterium tuberculosis infection.. J Immunol.

[23] Keane MP, Strieter RM (2002). The importance of balanced pro-inflammatory and anti-inflammatory mechanisms in diffuse lung disease.. Respir Res.

[24] Smith S, Liggitt D, Jeromsky E, Tan X, Skerrett SJ (2002). Local role for tumor necrosis factor alpha in the pulmonary inflammatory response to Mycobacterium tuberculosis infection.. Infect Immun.

[25] Zganiacz A, Santosuosso M, Wang J, Yang T, Chen L (2004). TNF-alpha is a critical negative regulator of type 1 immune activation during intracellular bacterial infection.. J Clin Invest.

[26] Zakharova M, Ziegler HK (2005). Paradoxical anti-inflammatory actions of TNF-alpha: inhibition of IL-12 and IL-23 via TNF receptor 1 in macrophages and dendritic cells.. J Immunol.

[27] Yamada H, Udagawa T, Mizuno S, Hiramatsu K, Sugawara I (2005). Newly designed primer sets available for evaluating various cytokines and iNOS mRNA expression in guinea pig lung tissues by RT-PCR.. Exp Anim.

[28] Betts MR, Nason MC, West SM, De Rosa SC, Migueles SA (2006). HIV nonprogressors preferentially maintain highly functional HIV-specific CD8+ T cells.. Blood.

[29] Darrah PA, Patel DT, De Luca PM, Lindsay RW, Davey DF (2007). Multifunctional TH1 cells define a correlate of vaccine-mediated protection against Leishmania major.. Nat Med.

[30] Beveridge NE, Price DA, Casazza JP, Pathan AA, Sander CR (2007). Immunisation with BCG and recombinant MVA85A induces long-lasting, polyfunctional Mycobacterium tuberculosis-specific CD4+ memory T lymphocyte populations.. Eur J Immunol.

[31] Pathan AA, Sander CR, Fletcher HA, Poulton I, Alder NC (2007). Boosting BCG with recombinant modified vaccinia ankara expressing antigen 85A: different boosting intervals and implications for efficacy trials.. PLoS ONE.

[32] Jain S, Kaushal D, DasGupta SK, Tyagi AK (1997). Construction of shuttle vectors for genetic manipulation and molecular analysis of mycobacteria.. Gene.

[33] Dhar N, Rao V, Tyagi AK (2000). Recombinant BCG approach for development of vaccines: cloning and expression of immunodominant antigens of M. tuberculosis.. FEMS Microbiol Lett.

[34] Mitchison DA, Wallace JG, Bhatia AL, Selkon JB, Subbaiah TV (1960). A comparison of the virulence in guinea-pigs of South Indian and British tubercle bacilli.. Tubercle.

[35] Allen SS, McMurray DN (2003). Coordinate cytokine gene expression in vivo following induction of tuberculous pleurisy in guinea pigs.. Infect Immun.

